# Supplementary material for: Global quantitative analysis of the human brain proteome and phosphoproteome in Alzheimer’s disease
Source: Sci Data. 2020 Sep 28;7:315. doi: 10.1038/s41597-020-00650-8 (PMC7522715; doi:10.1038/s41597-020-00650-8)
Supplement: Supplementary file 1 — Supplementary Material [file 41597_2020_650_MOESM1_ESM.pdf]

Supplementary Table of Contents

1) Supplementary Figure 1: Chromatography of off-line high-pH peptide separation. Page 1

2) Supplementary Figure 2: Evaluation of stability of quantification using two GIS samples. Page 2

3) Supplementary Table 1: Sample traits. Page 3

4) Supplementary Table 2: TMT channels arrangement. Page 4

a

Gradient of off-line fractionation

Time Interval (min)	Gradient (% B)	Flow rate (ml/min)
0	0.0	0.8
7.00	0.0	0.8
13.00	16.0	0.8
73.00	40.0	0.8
77.00	44.0	0.8
82.00	60.0	0.8
96.00	60.0	0.8

b

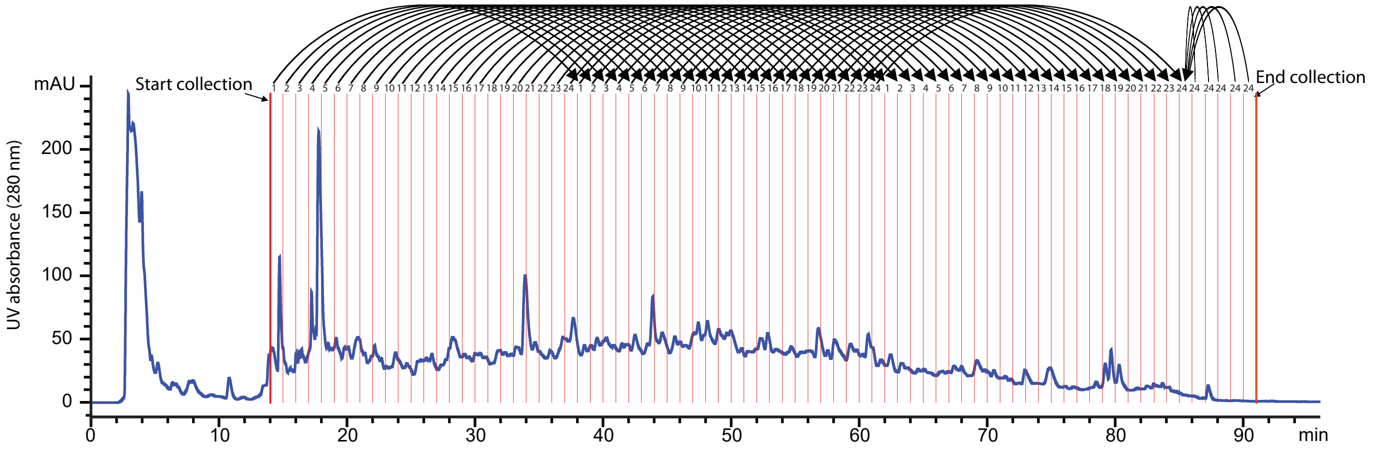

Supplementary Figure 1. Chromatography of off-line high-pH peptide separation. a) Chromatography gradient information. b) The TMT-labeled peptide mixture was separated on a 96 min gradient. In total, 77 fractions were collected from 14 to 91 minutes, with 1 fraction collected every min, which were further combined into 24 fractions. The 24 fractions were further combined into 12 fractions in an alternating manner (1 and 13, 2 and 14, etc.) for phosphoproteome analysis.

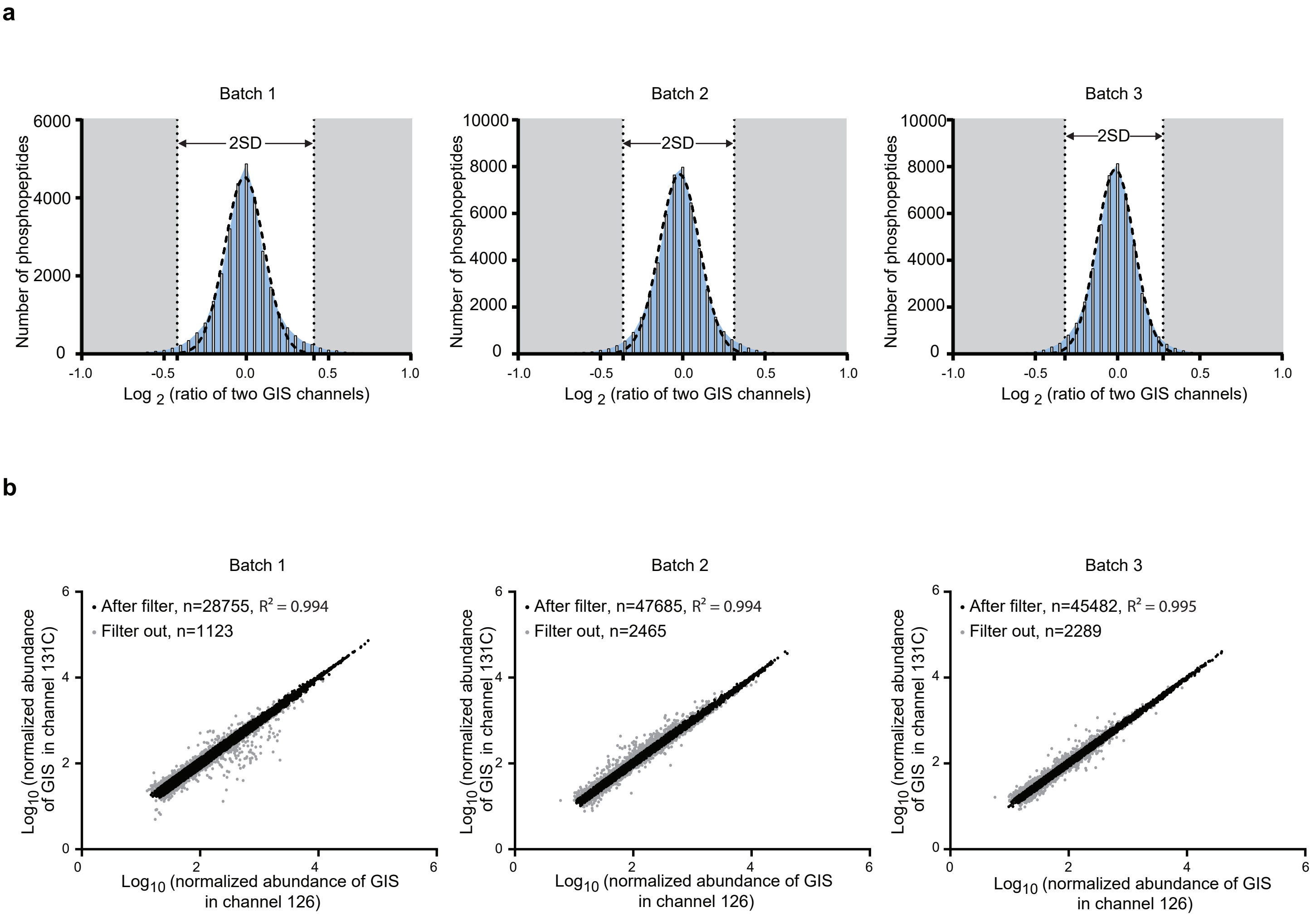

Supplementary Figure 2. Evaluation of stability of quantification using 2 GIS samples. (a) The Gaussian distribution of log2 ratios of the normalized abundance of 126/131C. The Gaussian curves were fit for all 3 batches using Graphpad Prism6. The peptides with log2(126/131C) ratios >2 standard deviation (SD) from 0 are highlighted (grey). (b) The adjusted correlation of normalized abundance from 126 and 131C channels after log2 ratio of (normalized abundance of 126/131C) data were filtered with 2 standard deviations. Those peptides that were >2 SD (grey points) were excluded from further analyses, while peptides with ≤2 SD were included (black dots). After filtering out variable peptides from the IMAC proteome, the GIS channels were better correlated.

Supplementary table 1. Sample traits

ID	sample	Batch	Channel	Diagnosis	CERAD	Braak	PMI (hr)	Age	ApoE	Race	Sex
GIS	-	1	126	GIS	NA	NA	NA	NA	NA	NA	NA
ASYM.OS02.270.127N.B1	OS02-270	1	127N	AsymAD	2	1	20.5	87	E3/4	3	male
AD.OS00.11.127C.B1	OS00-11	1	127C	AD	3	5	4	55	E3/3	3	male
AD.E06.155.128N.B1	E06-155	1	128N	AD	3	6	6.5	67	E2/3	3	male
ASYM.E15.97.128C.B1	E15-97	1	128C	AsymAD	3	4	5.5	80	E3/4	3	male
ASYM.OS03.394.129N.B1	OS03-394	1	129N	AsymAD	3	3	5	87	E2/3	3	female
CTL.A87.50.129C.B1	A87-50	1	129C	control	0	1	10	66	E3/3	2	male
AD.OS00.32.130N.B1	OS00-32	1	130N	AD	3	6	3.5	62	E3/4	3	male
CTL.A86.46.130C.B1	A86-46	1	130C	control	0	1	6	65	E3/3	3	male
CTL.E05.130.131N.B1	E05-130	1	131N	control	0	0	3	52	E3/4	3	female
GIS	-	1	131C	GIS	NA	NA	NA	NA	NA	NA	NA
GIS	-	2	126	GIS	NA	NA	NA	NA	NA	NA	NA
ASYM.E05.54.127N.B2	E05-54	2	127N	AsymAD	3	3	17	64	E4/4	3	female
ASYM.E05.184.127C.B2	E05-184	2	127C	AsymAD	2	2	20	81	E3/3	3	male
CTL.E08.145.128N.B2	E08-145	2	128N	control	0	0	28	45	E3/3	3	female
AD.OS00.12.128C.B2	OS00-12	2	128C	AD	3	6	6	72	E3/4	3	male
CTL.OS00.06.129N.B2	OS00-06	2	129N	control	0	1	8	60	E3/4	1	female
AD.E05.87.129C.B2	E05-87	2	129C	AD	3	6	4	61	E3/4	3	male
AD.OS98.11.130N.B2	OS98-11	2	130N	AD	3	6	6	65	E4/4	3	female
CTL.A93.03.130C.B2	A93-03	2	130C	control	0	1	4.5	70	E3/3	2	male
ASYM.E04.74.131N.B2	E04-74	2	131N	AsymAD	3	4	35.5	76	E2/4	3	male
GIS	-	2	131C	GIS	NA	NA	NA	NA	NA	NA	NA
GIS	-	3	126	GIS	NA	NA	NA	NA	NA	NA	NA
CTL.E08.101.127N.B3	E08-101	3	127N	control	0	2	11.5	78	E3/3	3	female
CTL.E06.41.127C.B3	E06-41	3	127C	control	0	2	10	57	E3/3	3	male
AD.E08.53.128N.B3	E08-53	3	128N	AD	3	6	8	78	E3/3	3	female
ASYM.OS94.54.128C.B3	OS94-54	3	128C	AsymAD	3	4	17.5	96	E3/3	3	female
AD.OS03.163.129N.B3	OS03-163	3	129N	AD	3	6	4.5	55	E3/4	3	female
ASYM.E13.49.129C.B3	E13-49	3	129C	AsymAD	3	4	19	89	E3/3	3	male
CTL.OS03.299.130N.B3	OS03-299	3	130N	control	0	2	6	69	E3/3	3	male
CTL.OS02.35.130C.B3	OS02-35	3	130C	control	0	1	6	75	E3/3	3	female
AD.E04.186.131N.B3	E04-186	3	131N	AD	3	6	7	72	E3/4	3	female
GIS	-	3	131C	GIS	NA	NA	NA	NA	NA	NA	NA

GIS represents Global Internal Standard.
CERAD represents Consortium to Establish a Registry for Alzheimer’s Disease, which used to evaluate the amyloid-beta plaque level.
Braak stage is used to describe the neurofibrillary tangle status in post-mortem tissues.
PMI repesents postmortem interval, which is defined as the length of time between death and corpse discovery
In Race section, 1 for Black or African American; 2 for Native Hawaiian or Other Pacific Islander; 3 for White.

Supplementary Table 2. TMT channels arrangement.

Batch	127N	127C	128N	128C	129N	129C	130N	130C	131N
1	OS02-270	OS00-11	E06-155	E15-97	OS03-394	A87-50	OS00-32	A86-46	E05-130
2	E05-54	E05-184	E08-145	OS00-12	OS00-06	E05-87	OS98-11	A93-03	E04-74
3	E08-101	E06-41	E08-53	OS94-54	OS03-163	E13-49	OS03-299	OS02-35	E04-186

Control AsymAD AD
